# Appraisal of patient-reported outcome measures in analogous diseases and recommendations for use in phase II and III clinical trials of pyruvate kinase deficiency

**DOI:** 10.1007/s11136-018-2025-y

**Published:** 2018-11-19

**Authors:** M. S. Salek, T. Ionova, J. R. Johns, E. N. Oliva

**Affiliations:** 1 School of Life and Medical Sciences, University of Hertfordshire, College Lane, Hatfield, AL10 9AB UK; 2 University Clinic St. Petersburg State University and Multinational Centre for Quality of Life Research, St. Petersburg, Russia; 3 Institute for Medicines Development, Cardiff, UK; 4 Haematology Unit, Grande Ospedale Metropolitano Bianchi Melacrino Morelli, Reggio Calabria, Italy

**Keywords:** Pyruvate kinase deficiency, Patient-reported outcome, PRO, Quality of life

## Abstract

**Purpose:**

Pyruvate kinase deficiency (PKD) is a rare disease and understanding of its epidemiology and associated burden remains limited. With no current curative therapy, clinical manifestations can be life threatening, clinically managed by maintaining adequate hemoglobin levels through transfusion and subsequent support, but with frequent complications. Treatment goals are to maintain/improve the patient’s quality of life. With new therapies, reliable, valid, and relevant patient-reported outcome (PRO) tools are required for use in clinical trials.

**Methods:**

Systematic literature search identified no current PRO tools for capturing/measuring the impact of PKD and treatments in clinical trials. Therefore, the search strategy was revised to consider conditions analogous to PKD in terms of symptoms and impacts that might serve as parallels to the experience in PKD; this included sickle cell anemia, thalassemia, and hemolytic anemia. Psychometric properties, strengths, and weakness of selected appropriate PRO instruments were compared, and recommendations made for choice of PRO tools.

**Results:**

In adult populations, EORTC QLQ C30 and SF-36v2 are recommended, the former being a basic minimum, covering generic HRQoL, and core symptoms such as fatigue. In pediatric populations, PedsQL Generic Core Scale to measure HRQoL and PedsQL MFS scale to measure fatigue are recommended.

**Conclusions:**

Some symptoms/life impacts may be unique to PKD and not observable in analogous conditions. A ‘Physico-Psychosocial Model’ derived from the ‘Medical Model’ is proposed to form the basis for a hypothesized conceptual framework to address the development of PKD-specific PRO instruments.

**Electronic supplementary material:**

The online version of this article (10.1007/s11136-018-2025-y) contains supplementary material, which is available to authorized users.

## Introduction

Pyruvate kinase deficiency (PKD) is the most common cause of chronic non-spherocytic hemolytic anemia (CNSHA), and is inherited as an autosomal recessive trait [1]. PKD is regarded as a rare disease, with gene frequency studies estimating prevalence of 1 per 20,000 persons in the white population [2]. Understanding of the epidemiology and burden associated with PKD remains limited, due to the low incidence of the condition. Clinical manifestations of the condition may include chronic mild or fully compensated anemia to life-threatening neonatal hemolysis requiring exchange transfusions and subsequent transfusion support [3]. Neonatal jaundice is common. In adults, the degree of anemia seems to be relatively constant, with exacerbations during acute infections and pregnancy [3]. Consequently, the disease is associated with significant negative impact on a patient’s quality of life (QoL). Thus, frequent complications of PKD include aplastic crises, extramedullary hematopoiesis, pregnancy complications of affected mothers, and need for exchange transfusion in the newborn period. Increasing severity of PKD (based on frequency of transfusion) has been associated with a trend of increasing ferritin and liver iron concentration [4].

There is currently no curative therapy for PKD, and the condition is clinically managed by maintaining adequate hemoglobin levels through transfusion and using splenectomy for patients with severe anemia or symptomatic hypersplenism [1, 5–7], both resulting in a marked increase of patient morbidity and mortality. Although hematopoietic stem cell transplantation may offer a cure for PKD, this has yet to demonstrate favorable benefit-risk balance, and is currently not standard practice [8]. Iron overload, in part due to chronic blood transfusions, often necessitates treatment with iron chelation therapy, which has been reported to result in various complications in nearly a quarter of patients receiving chelation [9]. Thus, the treatment goal for PKD is to maintain/improve the patient’s QoL.

An investigational therapy of AG-348, a small molecule allosteric activator of WT red cell pyruvate kinase, which directly addresses the underlying pathology of PK deficiency, is undergoing clinical development [10]. This new agent has potential to significantly improve a patient’s QoL. In addition, gene therapy offers promise as a safe and efficient treatment modality [11–14], similar to that of genetic correction for other diseases such as* β*-thalassemia in humans [15].

With the advancement of new therapies and trials, there is thus a need to identify reliable, valid, and relevant patient-reported outcome (PRO) tools for use in planned clinical trials. This paper presents a systematic search of the literature to identify current PRO tools that may be used to capture/measure the impact of PKD and its treatment in clinical trials. It aimed to (1) identify appropriate PRO instruments, based on analogous diseases with similar symptoms, and compare their psychometric properties, strengths, and weaknesses; (2) build a ‘Physico-Psychosocial Model’ for PKD; and (3) provide recommendations for the choice of PRO tools for use in future clinical trials.

## Materials and methods

The approach employed in this study involved three steps. First, understanding how patients experience symptoms and impacts associated with PKD. Second, developing a hypothetical conceptual framework for relevant PRO concepts in PKD and, finally, identifying the appropriate PRO measures for assessing such concepts.

**Fig. 1:**
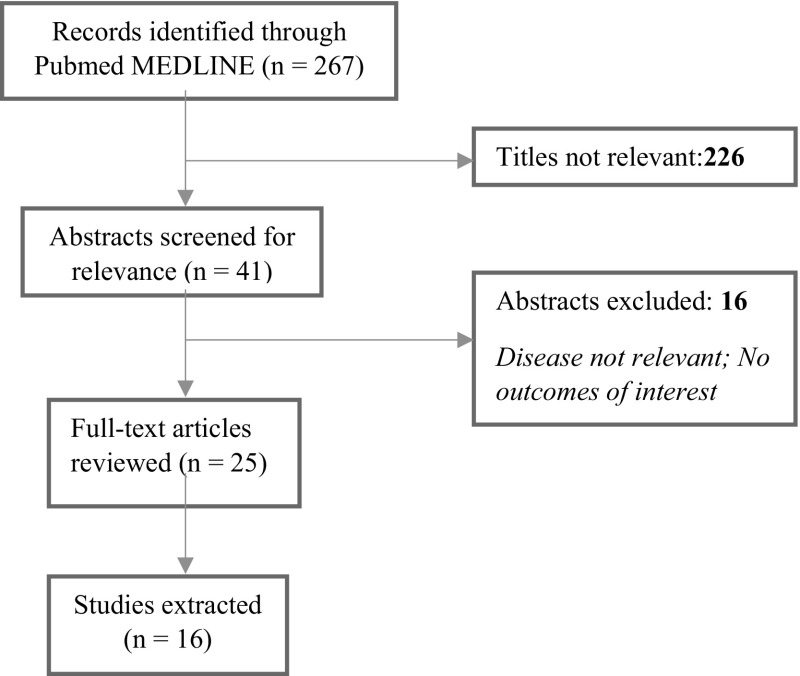
Flow diagram of studies in the literature review process, for the revised searches based on conditions analogous to pyruvate kinase deficiency

The literature searches were performed in two stages. Initially, searches were carried out for literature reporting results on PROs in PKD. Secondly, studies reporting key evidence on the development or validation of the most frequently used PRO measures were searched. Initial MEDLINE searches for studies reporting PROs in PKD (using the search terms in Table 1, block 1 and block 2) yielded no relevant results, and therefore the search strategy was revised to consider conditions analogous to PKD in terms of symptoms and impacts, which might serve as parallels to the experience in PKD; this included sickle cell anemia, thalassemia, and hemolytic anemia. Records identified, screened, excluded and included, and extracted for final analysis are shown in the flow chart in Fig. 1.

**Table 1:** Key terms used for literature searches

Block 1	Pyruvate kinase deficiency
Block 2	Quality of life; functional status; health status; functional impairment; functional limitations; activities of daily living

Thalassemia, sickle cell anemia, and PKD all give rise to splenomegaly, which results in symptoms of abdominal pain [16], chest pain [16], and anemia due to accompanying cytopenia in these diseases. All three diseases also give rise to hemolytic anemia resulting in symptoms of fatigue [17] and shortness of breath [18], sleep disturbances [19, 20], and jaundice [16, 17]. As a result of blood transfusions used as a treatment in these three diseases, iron overload occurs, resulting in heart [21, 22] or liver disease [22, 23], infections [16], and osteoporosis [24, 25]. Leg ulcers are another complication of all three diseases [16, 21, 26]. Finally, thalassemia can give rise to bone deformities [27], sickle cell disease to avascular necrosis of the bone [28], and PKD to joint pain [16]. It is these symptoms and their impacts that reduce patients’ reported reduction in quality of life, and thus it is very appropriate to use these analogous diseases to serve as parallels to the experience in PKD.

Following the development of a hypothetical conceptual framework and the identification of possible PRO measures, pragmatic searches were then carried out to identify psychometric attributes of the identified PROs. A short description of each tool is given in the supplementary data.

## Results

### Symptoms and health-related quality of life issues

No studies were found reporting symptoms or life impacts in PKD, except for a protocol of an ongoing observational study registered in a clinical trials database (https://clinicaltrials.gov). This study (NCT02053480), aimed at describing the range and symptoms, treatment, and complications related to PKD is underway in the USA at the Children’s Boston Hospital [29]. The study includes patients of all ages diagnosed with PKD, or patients with hemolytic anemia and a family member with genetically diagnosed PKD. The primary outcome was transfusion burden, while the secondary outcomes were patient-reported outcomes assessed on various measures such as the EuroQoL-5D-5L, Functional Assessment of Cancer Therapy-Anemia (FACT-An), Pediatric Quality of Life Inventory 4.0 (pedsQL 4.0), Pediatric Functional Assessment of Chronic Illness-Fatigue (pedsFACIT-F), and Patient-Reported Outcomes Measurement Information System Fatigue (PROMIS Fatigue).

Three qualitative studies describing the experience of patients with sickle cell disease (SCD) and patients receiving infusion iron chelation therapy provided insights into the possible patient-reported outcomes (PROs) in PKD. In addition, results from patients with chronic kidney disease (CKD)-related anemia are reported.

In another qualitative study, Stegenga et al. [30] explored the QoL of children with SCD aged 6–12 years (*n* = 10) receiving chronic transfusion therapy for stroke, using semi-structured interviews. Five themes reflecting the concerns of the children emerged from their data, including physical and psychological pain; school issues, i.e., attendance and being treated differently by teachers and peers; disease knowledge; transfusion therapy; and having a stroke. The authors mentioned that the children did not distinguish between disease and treatment-related aspects. In addition, given the sample size it is unclear whether these findings are generalizable.

Further, the experiences of adolescents and young adults with SCD, aged 15–35 years, have been reported by Thomas and Tylor [31], based on focus groups with patients (*n* = 17). Patients reported impacts in six major areas including growing up with SCD, education, the recurrent nature of the disease, employment, relationship, and hospitalization. The authors concluded that the aspects of life affecting SCD were equivalent to the core domains of the multidimensional WHOQOL including physical, psychological, social, and occupational and well-being, as well as levels of independence and environment.

In addition to direct disease impacts, complications such as iron overload are also of particular interest, especially due to the adverse consequences of iron chelation therapy. The QoL impacts of iron overload and infusion iron chelation therapy have been investigated by Abetz and colleagues [32] based on literature review and interviews with patients and experts.

The authors found no literature focusing on the impact of iron overload or iron chelation therapy on QoL, although this was reported incidentally in a few papers. In one study, 50% of patients reported that daily activities were negatively affected (prevented) due to desferoxamine treatment. Iron chelation therapy (ICT) with desferoxamine was associated with a degree of discomfort resulting in limitations in daily life activities. Characteristics of the infusion such as higher frequency also resulted in HRQoL impairment.

Results from the interviews with patients (thalassemia, sickle cell disease, myelodysplastic syndromes) performed by Abetz et al. [32] were consistent with the current literature as reported above. HRQoL impact was particularly profound where treatment started from a young age and persisted throughout life, for example in thalassemia. In addition to effects experienced by the patients, the impacts on family/parents of pediatric patients included parental stress, related to daily inserting of a needle into their child, and strain on the relationship between parent–child (family–patient). Adolescents experienced impacts on self-image, were unable to wear certain clothes, and felt embarrassed to go out as a result of bumps/bruises on their skin. Adults reported impacts on work, sex life, and social life. The impact of ICT on QoL was noted to be greatest among adolescents and young adults. These results suggest that infusion ICT has substantial impacts across multiple QoL domains. It is still unclear whether similar impacts are experienced in patients treated with other ICT.

Lasch et al. [33] investigated how adults with chronic kidney disease on dialysis experienced the symptoms of anemia, using semi-structured interviews with 55 patients. Their data revealed 20 major themes including energy, tiredness, endurance, shortness of breath, strength/weakness, and needing more sleep. Themes associated with the impacts of anemia included need to move slower; feeling bad/better; restless sleep; and effort required for exercise. Lasch and colleagues commented that all symptoms/impacts reported by the patients could be tied to a symptom cluster related to energy. The authors further mentioned that the patients related the energy symptoms cluster to rising and falling of hematocrit levels.

Further, in addition to the qualitative evidence presented above, five studies applying quantitative methods, using generic HRQoL measures such as the SF-36, EORTC QLQ C30, FACIT-fatigue, and PedsQL Generic Core Scale, showed reduced HRQoL across multiple domains, and increased levels of fatigue.

McClish et al. [34] assessed QoL in adults with SCD (*n* = 308) participating in the Pain in Sickle Cell Epidemiology Study (PiSCES) in the US. SF-36 scores indicated worse HRQoL relative to US national norms on all subscales except mental health. In comparison with dialysis patients, adults with SCD scored similarly on physical role and emotional role function, social functioning and mental health, worse on bodily pain, general health and vitality, and better on physical functioning. In comparison with people with cystic fibrosis, adults with SCD showed worse SF-36 scores on all subscales except mental health.

Dampier et al. [35] investigated HRQoL in children with SCD using data from the Collaborating Project of the Comprehensive Sickle Cell Centers (CSCC) clinical trial consortium, employing the PedsQL scales (Generic Core scale/Multidimensional Fatigue scale). Their results showed a progressive decline in all parent-reported PedsQL scale scores (physical, emotional, school functioning, total HRQoL, general fatigue, sleep/rest fatigue, total fatigue) across younger to older age groups (except for cognitive fatigue). Similar decline was observed in child-reported PedsQL scale scores (physical, emotional, social, school functioning, sleep/rest fatigue, cognitive fatigue scales). Parents and children differed on the rating of social functioning and cognitive fatigue, and on the overall, PedsQL scores; child reports were higher than parent reports. In comparison with age-matched healthy children, children with SCD showed much lower PedsQL scores (lower HRQoL and higher fatigue). The authors attributed the gradual but significant decline in HRQoL to acute and chronic complications (such as vaso-occlusive pain or priapism) which increased in frequency over time.

Ameringer et al. [36] recently described fatigue and its key biological and behavioral correlates, as well as its relationship with HRQoL, in adolescents and young adults with SCD (*n* = 60) aged 15–30. The majority of adolescents and young adults (69%) reported feeling unusually tired or fatigued in the previous week, and scores of various fatigue scales (MFSI-SF, PROMIS Fatigue) showed mild to moderate fatigue. Levels of fatigue did not differ by disease severity or biomarkers of inflammation. The correlation of hemoglobin and fatigue depended on the scale used for measuring fatigue. Higher fatigue was correlated (*r* = 0.31–0.70) with increased anxiety, pain, and sleep disruptions. Fatigue scales showed a moderate correlation with all subscales of the SF-36 (*r* = − 0.34 to − 0.74).

In thalassemia, HRQoL impairment similar or worse than that in SCD has been reported due to need for chronic transfusion and chelation therapy. A study by Sobota et al. [37] compared HRQoL reported in the Thalassemia Clinical Research Network’s Longitudinal Cohort (TLC) (*n* = 264, age ≥ 14) to US norms and published literature. In comparison to the US norms, TLC patients showed worse HRQoL on five of eight SF-36 subscales (physical functioning, role-physical, general health, social functioning, and role-emotional) and on both summary scales (physical and mental components). The greatest effect was in general health and physical domains. Women, older patients, and those with more disease complications and side effects from chelation reported lower HRQoL.

Further, Sobota and colleagues [37] argued that their results were similar to results obtained from two earlier studies of Payne et al. [38, 39] which reported lower age/gender-matched scores for all SF-36 domains in patients with thalassemia in comparison with UK norms. However, Sobota et al. [37] reported contrasting results from two studies from Italy, one reporting that scores in thalassemia patients were not much different from Italian SF-36 score norms. The other study reported that patients showed lower scores only for social functioning, role-emotional and mental component summary. Sobota and colleagues [37] argued that the older age of patients in the Italian studies explains the contrasting results.

Schrezenmeier et al. [40] reported the initial findings from the international Paroxysmal Nocturnal Haemoglobinuria (PNH) registry observational study (*n* = 1610) to describe clinical characteristics and disease-associated comorbidities in this patient population. Half of the patients were diagnosed with bone marrow disorder including aplastic anemia, hypoplastic anemia, myelodysplastic syndromes, myelofibrosis, and/or acute myeloid leukemia. The most common symptoms included fatigue (80%), dyspnea (64%), headache (63%), hemoglobinuria (62%), abdominal pain, scleral icterus, erectile dysfunction, chest pain, confusion, and dysphagia. Scores for all EORTC QLQ C30 domains were statistically significantly lower (i.e., lower HRQoL) for patients who had reported a clinical symptom of abdominal pain, chest pain, confusion, dysphagia, dyspnea, erectile dysfunction, fatigue, headache, hemoglobinuria, or scleral icterus in the six months prior to completing the baseline questionnaire compared with patients who had not experienced the symptoms.

### Physico-psychosocial model for pyruvate kinase deficiency

**Fig. 2:**
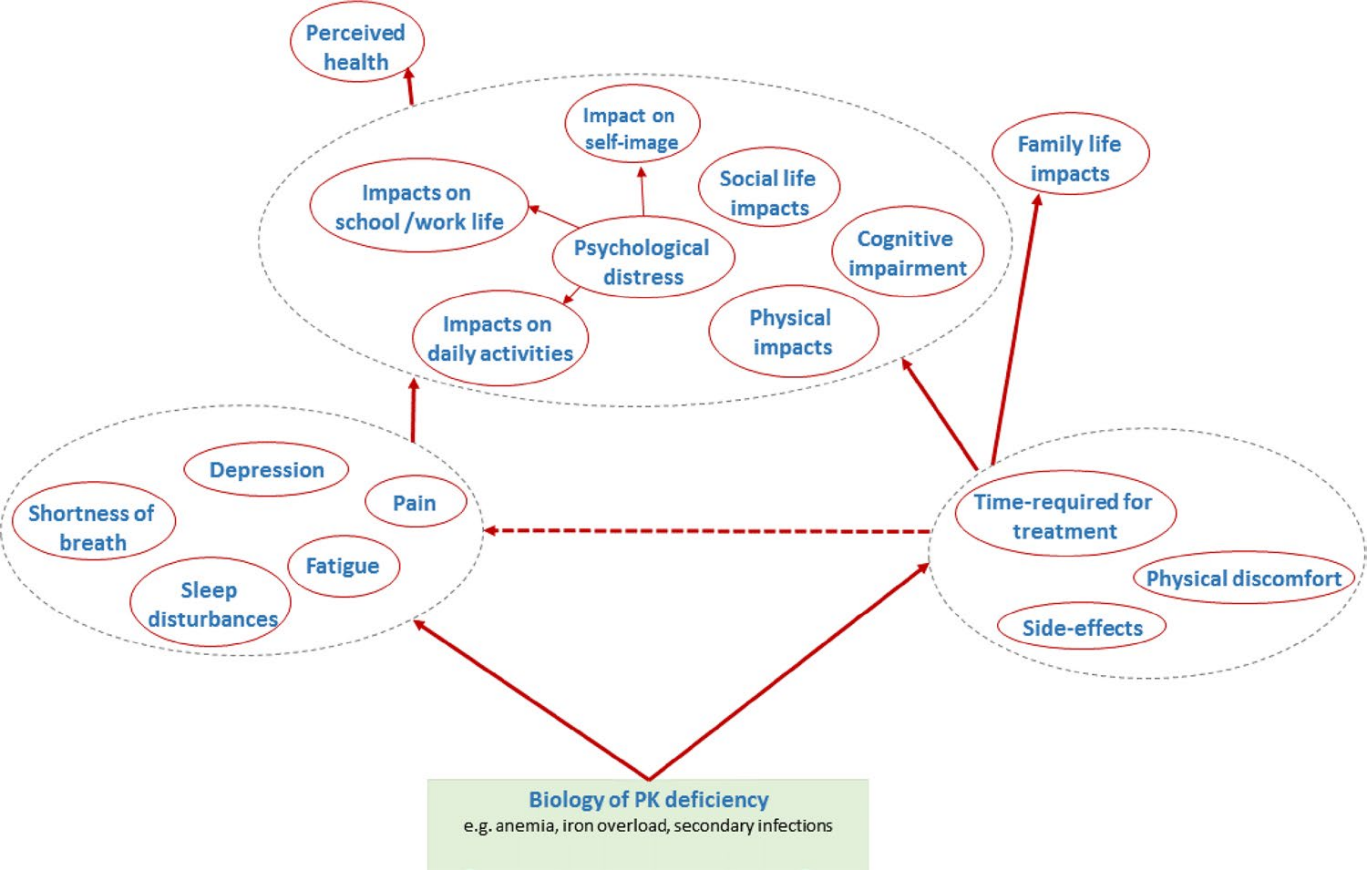
Pyruvate kinase deficiency physico-psychosocial model

Based on the evidence of symptoms and impairment in PKD analogous conditions, the possible patient-reported symptoms and impacts expected in PKD were hypothesized (Fig. 2).

Based on the major clinical features of PKD, i.e., anemia, iron overload, acute infections, and splenomegaly, the broad areas of patient outcomes include symptoms, HRQoL impacts, treatment-related impacts, and family life impacts. The key symptoms in PKD are likely to be related to anemia, jaundice, and splenomegaly, and include fatigue, sleep disturbances, abdominal pain, chest pain, shortness of breath, and depression. The requirement for chronic treatment, e.g., chronic blood transfusion, and iron chelation, from an early age is associated with substantial treatment burden, which includes the time/effort required to manage treatments, the physical discomfort associated with treatment, and the side effects of treatment. Ultimately, disease symptoms and treatment burden result in impairment in multiple domains of HRQoL including daily activities, psychological functioning, self-image, physical functioning, and social life. The substantial impacts on HRQoL and the treatment burden, consequently, have an impact on the family as a whole (these may be exaggerated for pediatric patients but are otherwise equally important for all patients).

### HRQoL measures

The most frequently used measures in PKD/PKD analogous conditions that address concepts in the hypothesized Physico-Psychosocial model outlined in Fig. 2 were searched. A total of 11 PRO measures, 1 disease-specific measure, and 1 generic family life impact measure were identified. The EQ-5D-5L was excluded as it was considered to be more appropriate for evaluating utilities for the purposes of economic evaluation, and less fitting for capturing treatment effect in trial settings, and FACIT-Fatigue excluded as a stand-alone measure, as its contents are already included as the fatigue scale in the FACT-An. Details of each measure are given in the Supplementary Data.

The PRO measures were evaluated against standards published in the FDA PRO guidance [41]. In brief, the adequacy of any PRO measure to support medical product labeling claims depends on its characteristics, conceptual, content validity, and measurement properties. A comparison of the content coverage is reported in Table 2, while an evaluation of the psychometric attributes is presented in Table 3.

**Table 2:** Similarities and differences in conceptual coverage of health-related quality of life instruments used in diseases analogous to PK deficiency

Health status/HRQoL domains/symptoms	WHOQOL-Bref [42]	SF-36v2 [43]	EORTC QLQ C30 [44, 45]	CHQ [46, 47]	PedsQL [46, 48]	PedsQL MFS [49, 50]	PedsFACIT-F [51]	PedsQL SCD [52]	ASCQ-Me [53]	FROM-16 [54]
Physical well-being	●	●	●	●	●			**●**	●	
Psychological distress	●	●	●	●	●				●	
Impact on daily activities	●	●	●	●	●			●		
Impact on school/productivity				●	●					
Impact on work/productivity	●	●	●							
Impact on self-image	●			●					●	
Impact on social life	●	●	●	●	●				●	
Perceived general health/HRQoL	●	●	●	●						
Cognitive impairment	●		●						●	
Family life impacts			●	●						●
Fatigue		●	●			●	●			
Shortness of breath			●							
Pain (abdomen, chest, headache)	●	●	●	●				●	●	
Sleep disturbance			●						●	
Depression	●	●	●							

In general, a concept was considered to have been addressed by an item if there was at least 1 item addressing that concept in a particular PRO measure

*SF-36* short-form medical outcome, *WHOQOL-BREF* WHO Quality of Life-BREF, *FACT-An* functional assessment of cancer therapy-anemia, *FROM-16* Family-Reported Outcome Measure-16, *PedsQL* Pediatric Quality of Life Inventory, *MFS* Multidimensional Fatigue Scale, *pedsFACIT-F* pediatric Functional Assessment of Chronic Illness Therapy-Fatigue, *CHQ* Children’s Health Questionnaire, *ASCQ-Me* adult sickle cell quality of life measurement information system, *FROM* family-reported outcome measure

**Table 3:** Evaluation of psychometric attributes of PRO measures

	Content validity	Construct validity	Internal consistency	Test–retest	Responsiveness	MCID	Respondent burden; completion time	Language translations
SF-36	0	++	++	0	+	0	Minimal; 5–10 min	> 170 countries
WHOQOL-BREF	++	++	++	+	0	0	Minimal; < 5 min	> 23 countries (cultural adaptations)
FACT-An	++	++	++	++	++	++	Minimal; 5–15 min	> 45 languages^a^
EORTC QLQ C30	++	++	++	++	++	++	Minimal 5–15 min	> 80 languages
FROM-16	++	++	++	++	++	−	Minimal; < 3 min	2 languages
PedsQL 4.0 Generic Core Scale	+	++	++	++	++	++	Minimal; < 4 min	> 21 languages
PedsQL 4.0 MFS	+	++	++	++	++	++	Minimal; < 4 min	> 21 languages
PedsQL SCD	++	++	++	++	++	++	Minimal; < 4 min	–
pedsFACIT-F	++	++	++	−	−	++	5–10 min	–
CHQ	0	++	++	0	0	0	5–15 min	> 72 languages
ASCQ-Me	++	++	++	−	−	−	~ < 10 min	–

*SF-36* short-form medical outcome, *WHOQOL-BREF* WHO Quality of Life-BREF, *FACT-An* functional assessment of cancer therapy-anemia, *EORTC QLQ C30* European Organisation for the Research and Treatment of Cancer Quality of Life Questionnaire Core 30, *FROM-16* Family-Reported Outcome Measure-16, *PedsQL* Pediatric Quality of Life Inventory, *MFS* Multidimensional Fatigue Scale, *SCD* sickle cell disease, *pedsFACIT-F* pediatric Functional Assessment of Chronic Illness Therapy-Fatigue, *CHQ* Children’s Health Questionnaire, *ASCQ-Me* adult sickle cell quality of life measurement information system, ++: adequate data available from PK-d analogous population, +: data are available from PK-d analogous population, although inadequate, 0: data available in other patient populations, but not from PK-d analogous populations, −: data not reported in any patient population

^a^For the FACT-G and FACT-F subscales

### Appraisal of HRQoL measures

#### Adults

Four most relevant QoL/HRQoL measures used in patient populations analogous to PKD patients, the WHOQOL-BREF, the SF-36, the EORTC QLQ C30, and the FACT-An were compared (Table 4). Of these four, the EORTC QLQ C30 addressed all impact as well as symptom concepts except for “self-image” (Table 2). The WHOQOL-BREF, although based on a more broad definition of health, has psychometric evidence that supports using this scale for adults with chronic conditions and addresses the individualized nature of HRQOL, so is appropriate for this purpose [55]. The SF-36 and the FACT-An (particularly the FACT-G subscale) address similar impact-related concepts, except for one concept “perceived general health/HRQoL” which is assessed in the SF-36 only. The WHOQOL-BREF and the SF-36 address most domains with greater detail in comparison with the FACT-An, i.e., with more items addressing a wider range of issues. For example, whereas the FACT-An contains 2 items directly related to work, the SF-36 has 7 items/2 subscales to represent this issue. The FACT-An covers four of the five key symptom concepts, excluding “depression”; the SF-36 addresses three symptom concepts, while the WHOQOL-BREF covers two only. The “shortness of breath” and “sleep disturbances” concepts have been excluded from the WHOQOL and SF-36.

**Table 4:** Appraisal of health-related quality of life measures

Attribute	Advantages	Disadvantages
SF-36	• The most widely used PRO measure in general in PKD analogous conditions	• Floor/ceiling effects for some subscales (e.g., PH) in SCD • Published dimensional structure not supported in SCD (a 3 component structure was reported in a Jamaican population) • Content validity in SCD not demonstrated
WHOQOL-BREF	• Cross-culturally developed	• Measures relatively broader and subjective QOL domains, which may be affected by other factors unrelated to PK-d treatment • Limited regulatory experience
FACT-An	• Extensive regulatory experience based on clinical trials of ESAs in cancer patients • Integrates generic HRQOL aspects as well as impacts/symptoms specific to anemia	
EORTC QLQ C30	• Has best coverage of impacts/symptom-related concepts for the psychosocial model • Extensive regulatory experience based on clinical trials of ESAs in cancer patients • Integrates generic HRQOL aspects relevant to anemia	
CHQ		• Domain structure is different between healthy children and those with a chronic disease • Use of multiple response scaling and recall periods has potential to be confusing
PedsQL	• Has the most extensive psychometric evidence in pediatric SCD • Has been used to support a PRO labeling claim for Soliris in PNH in Europe • Recall period and response scaling appropriate for pediatrics	
PedsQL MFS	• One of the most validated and widely used pediatric fatigue measures in SCD • Has been used to support a PRO labeling claim for Soliris in PNH in Europe	
PedsQL SCD	• Content is underpinned by extensive qualitative research across children 5–8 years, parents, and experts in SCD • Provides comprehensive measurement capabilities in combination with other PedsQL scales (i.e., generic, fatigue)	• As the measure is relatively new, current psychometric evidence is not extensive • Some scales could not discriminate between mild-severe disease for child report
pedsFACIT-F	• Superior measurement attributes—its development applied modern test theory • May permit possible comparison with scores from adult-version FACIT-F	• Some key psychometric properties have not been reported, e.g., responsiveness and test–retest reliability • Clinical trials using the measure were scarce • Regulatory uncertainty with use of PROs based on item banks in drug development
ASCQ-Me	• The only item bank for ASCQ-Me • High precision/strong measurement capabilities as a result of use of modern test theory as well as classical test theory	• Regulatory uncertainty with use of PROs based on item banks in drug development
FROM-16	• Rigorous development and validation across at least ten medical specialties • High precision/strong measurement capabilities as a result of use of modern test theory as well as classical test theory	• As the measure is quite new, use in clinical trials remains limited

Extensive psychometric evidence including construct validity, reliability, and responsiveness is available supporting the validity of the EORTC QLQ C30, SF-36v2, and FACT-An measures in PKD analogous conditions such as sickle cell and cancer-treatment-related anemia.

#### Pediatric/adolescents

Comparison of HRQoL measures used in pediatric patients considered the Children’s Health Questionnaire (CHQ) and the PedsQL 4.0 Generic Core Scales (Table 4). The CHQ appeared to have better coverage of impact-related concepts in comparison with the PedsQL 4.0, in particular the “impact on self-image,” “perceived general health/HRQOL,” and “family impacts.” For symptom-related concepts, the CHQ addresses one concept, while the PedsQL 4.0 Generic score includes none. Such an omission in the PedsQL Generic Core Scale may be due to the availability of a SCD-specific PedsQL module, which seemed to be the most validated SCD-specific instrument in the pediatric population (Table 4).

Although most subscales of the CHQ apply a recall period of 4 weeks, one subscale (change in health) uses a recall period of 1 year. Long recall periods are generally not recommended due to concerns about accuracy of recall and potential biases. On the other hand, as assessment of HRQoL occurs at a certain interval within a study, assessing differences between such “actual” snapshots of the patients’ conditions provides the most reliable way of assessing change over time.

For the assessment of fatigue, two measures, the PedsQL MFS and the pedsFACIT-F, were compared (Table 4). Both measures address multiple dimensions of fatigue including physical as well as psychological aspects, although the PedsQL MFS additionally includes sleep aspects. More psychometric evidence is available for the PedsQL MFS relative to the pedsFACIT-F; specifically evidence of the pedsFACIT was not available.

For family life impacts, the Family-Reported Outcome Measure (FROM-16) was the only measure considered across the pediatric, adolescent, as well as adult patient populations (Table 4). This measure has been validated for use across all disease areas, and in all patient populations. There is to date limited use of this measure in clinical trials, reflecting the novelty of the conceptualization of family life impact as a broad and generic concept. Other measures such as the Family Dermatology Life Quality Index (FDLQI) or the PedsQL Family Impact Module were not reviewed, as they were considered to be too narrowly focused on a specific disease/patient population.

## Discussion

The PRO instruments potentially useful in PKD trials have been identified and their psychometric properties, namely strengths and weaknesses, were compared. On this basis, recommendations for the choice of PRO tools for use in future clinical trials are provided here. The relevance of PRO measures for use in PKD trials has been evaluated based on the understanding that clinical trials may include adult patient populations as well as pediatrics and adolescents. These conclusions therefore address the outcome measurement’s unique requirements of these groups.

Relevant patient-reported outcomes (PROs) in PKD encompass disease-specific symptoms, generalized impacts on multiple HRQoL domains and impacts on the wider family; this is the case for pediatrics, adolescents, as well as adult patient populations. Therefore, all these areas of impact should be measured in Phase II and III trials. Thus, measures addressing generic HRQoL, disease-related symptoms (particularly anemia-related symptoms such as fatigue), and family impacts should be considered.

In the adult population, the uses of EORTC QLQ C30 and SF-36v2 are recommended, the former being the basic minimum, particularly because this measure covers all key impacts as well as symptom concepts in the hypothesized physico-psychosocial model, including generic HRQoL, as well as core symptoms such as fatigue. It would be beneficial to use SF-36v2 in a study to validate results from EORTC QLQ C30, as well as to facilitate further comparative research in PKD, with other similar/related diseases. The SF-36v2 would be a better additional generic PRO measure than WHOQOL-BREF based on extensive experience with the SF-36 in clinical trials across PKD analogous conditions and a lack of such evidence with the WHOQOL-BREF.

In the pediatric population, the uses of PedsQL Generic Core Scale and the PedsQL MFS scale are recommended. The PedsQL Generic Core Scale is recommended as a measure of HRQoL, over the CHQ. The PedsQL MFS scale is recommended as a measure of fatigue over the pedsFACIT.

For pediatrics, adolescents, as well as the adult patient populations, the FROM-16 is recommended for the measurement of family burden associated with PKD. At present, this is considered to be the only generic measure of family life impact of illness appropriate for use across different patient populations. For example, the PedsQL has a family life impact module; however, this may not be of use in an adult population. Inclusion of this measure may be more appropriate for Phase III trials.

As currently no PRO measure has been validated in PKD, we recommend exploratory qualitative research to generate required content validity evidence (in a form of a pilot study) to support the above-recommended measures in PKD. Furthermore, we recommend that the blinded data from the phase II trial be utilized to assess/confirm the validity, reliability, responsiveness, and MCID of the recommended measures in PKD.

Thalassemia and paroxysmal nocturnal hemoglobinuria are clearly analogous to PKD, but sickle cell disease is a much more complicated disorder. The pathophysiology of sickle cell disease, in particular vaso-occlusive crises, is very different from what occurs in PKD. However, the selected PRO tools measure the impact of the disease and treatments from patient’s perspectives, and the analogous diseases chosen have symptoms that will produce similar patient experiences, even if the pathophysiology of the disease is somewhat different and some symptoms are different.

A major limitation to the above recommendations is that it is not possible to determine which symptoms/life impacts seen in the PKD analogous conditions may actually be observed in patients with PKD. Similarly, it is likely that some symptoms/life impacts in PKD are unique to the disease, and are not observable in analogous conditions, for example aplastic crises, osteopenia/bone fragility, extramedullary hematopoiesis, post-splenectomy sepsis, pulmonary hypertension, and leg ulcers [56]. Therefore, it would be ideal, at the minimum, to obtain the views of therapeutic experts on key symptoms and life impacts. A patient diary may then be developed within a short-time framework, to address those symptoms/life impact not covered in the current PRO measures, based on expert input. Social networking communities of patients with PKD may also be used to rapidly develop and validate such a diary.

The developed Physico-Psychosocial model for PKD may be helpful in future studies with a PRO component in patients with PKD. It may also form the basis for a hypothesized conceptual framework to be used for the development of a disease-specific PRO tool to capture/measure the QoL and symptom impact of PKD and its treatments in clinical trials.

At this stage, it is not possible to use empirical testing, e.g., structural equation modeling (SEM)/path analysis on this Physico-Psychosocial model for PKD. As there is currently no tool in this population, the data that we used came from variety of non-PKD tools for analogous diseases, applied in different settings. Therefore, this model should be a starting point to guide the next step to develop a specific Pyruvate Kinase Deficiency tool. Then, through data collection using this tool in a PKD population, it will be possible to test and either confirm or refute this Physico-Psychosocial Model.

Additionally, as the data used for this review came from a range of tools for analogous diseases, with a high level of heterogeneity among the different studies, it does not lend itself to meta-analysis and determination of effect size. Although the Pyruvate Kinase Deficiency Natural History Study (PKD NHS, https://clinicaltrials.gov, NCT02053480), a longitudinal, multicenter, international patient registry, is collecting retrospective and current clinical information and patient-reported outcome measures at enrolment and annually (expected study completion Dec 2020), there are limited data from PRO studies on PKD and quality of life. Until more data are available, effect size remains unknown.

## Electronic supplementary material

Below is the link to the electronic supplementary material.

Supplementary material 1 (PDF 282 KB)

## Glossary

Allosteric activator: Binding of one ligand enhances the attraction between substrate molecules and other binding sites
Aplastic crises: Temporary cessation of red cell production
Autosomal recessive trait: Ways that a trait, disorder, or disease can be passed down through families whereby two copies of an abnormal gene must be present in order for the disease or trait to develop
*β*-Thalassemia: A genetic blood disorder that reduces the production of hemoglobin
Chelation Therapy: A form of complementary therapy involving circulating a chelating solution in the bloodstream to bind toxins
Chronic non-spherocytic hemolytic anemia: A group of rare, genetically transmitted blood disorders characterized by the premature destruction of red blood cells
Cytopenia: Reduction in the level of red blood cells
Desferoxamine: A medication that binds iron used to treat iron overload
Extramedullary hematopoiesis: Hematopoiesis (differentiation processes that lead to the formation of all blood cells from hematopoietic stem cells) occurring in organs outside of the bone marrow
Hematopoietic stem cell transplantation: Is the transplantation of multipotent hematopoietic stem cells
Hemolytic anemia: A form of anemia due to hemolysis, the abnormal breakdown of red blood cells
Myelodysplastic syndromes: A group of cancers in which immature blood cells in the bone marrow do not mature and therefore do not become healthy blood cells
Neonatal hemolysis: Hemolytic disease of the newborn
Neonatal jaundice: A yellowish discoloration of the white part of the eyes and skin in a newborn baby due to high bilirubin levels
Paroxysmal nocturnal hemoglobinuria: A rare blood condition where blood cells are prone to be attacked by part of the body’s immune system
Pathophysiology: Functional changes associated with or resulting from disease or injury
Post-splenectomy sepsis: Body’s response to infection causing injury to its own tissues and organs following removal of the spleen
Pulmonary hypertension: Increased blood pressure within the arteries of the lungs
Pyruvate kinase: The enzyme that catalyzes the final step of glycolysis
Structural equation modeling: A diverse set of mathematical models, computer algorithms, and statistical methods that fit networks of constructs to data and includes confirmatory factor analysis, path analysis, and partial least squares
Symptomatic hypersplenism: Exhibiting an overactive spleen, removing too many blood cells, including healthy ones
Path analysis: An extension of multiple regression used to provide estimates of the magnitude and significance of hypothesized causal connections between sets of variables and includes multiple regression analysis, factor analysis, and discriminant analysis
Vaso-occlusive crises: A common painful complication of sickle cell anemia where circulation of blood vessels is obstructed by sickled red blood cells, causing ischemic injuries
