# Supplementary material for: Appraisal of patient-reported outcome measures in analogous diseases and recommendations for use in phase II and III clinical trials of pyruvate kinase deficiency
Source: Qual Life Res. 2018 Nov 19;28(2):399–410. doi: 10.1007/s11136-018-2025-y (PMC6373289; doi:10.1007/s11136-018-2025-y)
Supplement: Supplementary file 1 — Supplementary material 1 (PDF 282 KB) [file 11136_2018_2025_MOESM1_ESM.pdf]

Short Form Health Survey (SF-36)

The Short Form health survey 36-item version 2 (SF-36v2) is a generic measure of health status/HRQoL developed for use in population surveys and across multiple health conditions (McDowell, 2006). The SF-36 includes 36 items using a recall period of 1 month. An additional item asking about change in general health over the last year is included.
The SF-36 has been validated across multiple health conditions. For PKD analogous conditions, psychometric evidence has been reported in thalassemia major (Persian version) (Jafari et al., 2008), and in sickle-cell disease (Jamaican sample) (Asnani et al., 2009b). The SF-36 showed responsiveness to change in beta-thalassemia patients treated with deferasirox in the EPIC clinical trial (Porter et al. 2012).

WHO Quality of Life -BREF (WHOQOL-BREF)

The WHOQOL-BREF is a 26-item measure for assessing subjective quality of life in adults, and is a brief version of the WHOQOL-100 questionnaire. The items use a 5-point Likert interval scale and a recall period of two weeks. Strong psychometric attributes have been reported for the WHOQOL-Bref in a population of patients representing multiple health conditions and countries (Skevington et al., 2004). Validity and reliability of the WHOQOL-BREF have been reported in patients with sickle cell disease (Asnani et al., 2009a). Responsiveness has been reported in other disease areas.

European Organisation for the Research and Treatment of Cancer Quality of Life Questionnaire Core 30 (EORTC QLQ C30)

The EORTC QLQ 30 was developed as a measure of HRQoL in patients with cancer for use in international clinical trials. The measure uses a modular system and includes a generic questionnaire and additional cancer-specific modules. The 30 items of the generic questionnaire use a recall period of one week, and apply a 4-point Likert or visual analogue scales for responses. Evidence of the validity and responsiveness of the EORTC QLQ C30 is extensive (Aaronson et al., 1993; Luckett et al., 2011), and this is the most frequently used cancer-specific HRQoL measure across various cancers e.g. hematological malignancies.

Functional Assessment of Cancer Therapy – Anemia (FACT-An)

The Functional Assessment of Cancer Therapy – Anemia (FACT-An) was developed as a measure of generic HRQOL, fatigue and anemia-related symptoms in people with anemia related to cancer. This measure includes 48 items; 28 items assess generic HRQOL (FACT-G scale), and the others are specific to fatigue (13 items) and anemia (7 items). Items are rated on a 4-point Likert scale, and apply a recall period of 7 days. The FACT-An has been validated in cancer patients with anemia (Celia, 1998). The FACT-An scores showed good responsiveness in > 14 clinical trials of erythropoiesis-stimulating agents (ESAs) in cancer patients (Bohlius et al., 2014). This measure is currently being used in the *Pyruvate Kinase Deficiency Natural History Study (PKD NHS)* (NCT02053480) being carried out by the Children's Hospital in Boston.

Children's Health Questionnaire (CHQ)

The Children's Health Questionnaire (CHQ) was developed as a measure of generic HRQOL in children 5 to 18 years old. The measure includes a self-report version with 87 items (for children 10 to 18 years), and proxy-report versions with 50 or 28 items. Items are scored on a 4 to 6-point Likert scale and use varying recall periods such as 4 weeks (for most items), 1 year (for 'change in health' subscale) and 'general' (for global health/general health perception/family cohesion subscale).

The CHQ has been extensively validated in children with chronic illnesses such as asthma and cancer (Hullmann & Ryan, 2011). However, psychometric information in PKD analogous conditions is limited; in particular validity and reliability of the CHQ has been reported in sickle-cell disease (Panepinto et al. 2004). The CHQ has demonstrated responsiveness to change in disease activity, with the psychosocial summary score showing less responsiveness.

Pediatric Quality of Life Inventory Scales (PedsQL)

PedsQL generic core scale

The PedsQL 4.0 scale was developed as a modular measure of HRQoL in children and adolescents aged 2 to 18 years, and consists of parallel child/adolescent self-report and a parent proxy report. This measure include 23 items assessed on a 5-point Likert scale and applies a recall period of 1 month. Psychometric evidence supporting the use of this measure in children across different disease areas is available (Hullmann & Ryan, 2011). Psychometric data in sickle cell patients is limited to validity and reliability (Panepinto et al., 2008). Other disease/domain specific modules are available such as the PedsQL Multidimensional Fatigue Scale and the PedsQL Sickle-Cell Scale, which are described below.

PedsQL Multidimensional fatigue scale (PedsQL MFS)

The PedsQL MFS is an 18-item measure developed for assessing fatigue in children/adolescents. The format, response scale and recall period are identical to the PedsQL™ 4.0 Generic Core Scales. The PedsQL MFS has demonstrated reliability, validity, and responsiveness in children with cancer (Tomlinson et al., 2013). The PedsQL MFS scores showed sensitivity to change following physical activity; but no change following treatment with chemotherapy in children with cancer (Tomlinson et al., 2013). Available psychometric evidence in PKD analogous conditions includes data on reliability, validity, and dimensional structure in sickle cell disease (Panepinto et al., 2014).

PedsQL Sickle cell disease (PedsQL SCD)

The PedsQL SCD is a 43-item measure developed for assessing sickle cell specific HRQoL. The formatting and layout of the measure is similar to other PedsQL scales; items use a 5-point Likert scale for responses and apply a 1-month recall period. Adequate reliability and construct validity have been reported for this scale (Panepinto et al., 2012), however evidence of responsiveness is still lacking.

Pediatric Functional Assessment of Chronic Illness Therapy- Fatigue (pedsFACIT-F)

The pedsFACIT-F is an 11-item measure of multi-dimensional fatigue. Evidence of its psychometric attributes remains limited. The reliability, validity and dimensional structure of the measure have been tested in cancer patients (at least half were anemic) (Lai et al., 2007). Psychometric evidence in patient populations from PKD analogous conditions is currently not available.

Adult Sickle Cell Quality of Life Measurement System (ASCQ-Me)

The ASCQ-Me is an item bank for assessing PROs in adults with SCD. The 92 items of the ASCQe-Me measure six impact areas/outcomes in SCD (Sleep, Pain, Stiffness, Cognitive, Emotional, and Social). Methodological rigor was similar to that employed in the development of the NIH PROMIS item banks. The dimensional structure of the item banks, reliability and construct validity of the ASCQ-Me have been established (Keller et al., 2014).

Family Reported Outcome Measure (FROM-16)

The family reported outcome measure (FROM-16) is a generic measure of the impact on the quality of life a family member or partner resulting from having a person in a family with an illness. The measure includes 16 simple questions relating to the current impact experienced by the family member or partner rated on a 3-point Likert scale. Psychometric evidence available for this measures is based on classical as well as modern test theory (Golics et al., 2013).

References.

Aaronson NK, Ahmedzai S, Bergman B, Bullinger M, Cull A, Duez NJ, Filiberti A, Flechtner H, Fleishman SB, de Haes JCJM, Kaasa S, Klee MC, Osoba D, Razavi D, Rofe PB, Schraub S, Sneeuw KCA, Sullivan M, Takeda F. 1993. The European Organisation for Research and Treatment of Cancer QLQ-C30: A quality-of-life instrument for use in international clinical trials in oncology. J Nat Cancer Inst. 85:365-376.

Asnani, M., Lipps, G. & Reid, M., 2009a. Utility of WHOQOL-BREF in measuring quality of life in Sickle Cell. Health Qual Life Outcomes. 7:75-80.

Bohlius J, Tonia T, Nüesch E, Jüni P, Fey MF, Egger M, Bernhard J. 2014. Effects of erythropoiesis-stimulating agents on fatigue- and anaemia-related symptoms in cancer patients: systematic review and meta-analyses of published and unpublished data. Br J Cancer 111(1):33-45.

Cella D. 1998. Factors influencing quality of life in cancer patients: anemia and fatigue. Semin Oncol. (3 Suppl 7):43-6.

Golics CJ, Basra MK, Finlay AY, Salek S. 2013. The development and validation of the Family Reported Outcome Measure (FROM-16)(©) to assess the impact of disease on the partner or family member. Qual Life Res. 23(1):317-26.

Hullmann S, Ryan J. 2011. Measures of general pediatric quality of life: Child Health Questionnaire (CHQ), DISABKIDS Chronic Generic Measure (DCGM), KINDL-R, Pediatric Quality of My Life Questionnaire (QoML). Arthritis Care Res (Hoboken). 63 Suppl 11:S420-30.

Jafari H, Lahsaeizadeh S, Jafari P, Karimi M. 2008 Quality of life in thalassemia major: reliability and validity of the Persian version of the SF-36 questionnaire. Journal of Postgraduate Medicine 54(4):273–5.

Keller SD, Yang M, Treadwell MJ, Werner EM, Hassell KL. 2014. Patient reports of health outcome for adults living with sickle cell disease: development and testing of the ASCQ-Me item banks. Health Qual Life Outcomes 12:125-135.

Lai JS, Cella D, Kupst MJ, Holm S, Kelly ME, Bode RK, Goldman S. 2007. Measuring fatigue for children with cancer: development and validation of the pediatric Functional Assessment of Chronic Illness Therapy-Fatigue (pedsFACIT-F). J Pediatr Hematol Oncol. 29(7):471-9.

Luckett T, King MT, Butow PN, Oguchi M, Rankin N, Price MA, Hackl NA, Heading G. 2011. Choosing between the EORTC QLQ-C30 and FACT-G for measuring health-related quality of life in cancer clinical research: issues, evidence and recommendations. Annals of Oncology 22(10):2179-2190.

McDowell, I., 2006. *Measuring Health: A Guide to Rating Scales and Questionnaires*, Oxford University Press.

Panepinto JA, O'Mahar KM, DeBaun MR, Rennie KM, Scott JP. 2004. Validity of the child health questionnaire for use in children with sickle cell disease. J Pediatr Hematol Oncol. 26(9):574-8.

Panepinto JA, Pajewski NM, Foerster LM, Hoffmann RG. 2008. The performance of the PedsQL generic core scales in children with sickle cell disease. J Pediatr Hematol Oncol. 30(9):666-73.

Panepinto, J.A., Torres, S. & Varni, J.W., 2012. Development of the PedsQL^{TM} Sickle Cell Disease Module items: qualitative methods. Qual Life Res. 21(2):341-57.

Panepinto JA, Torres S, Bendo CB, McCavit TL, Dinu B, Sherman-Bien S, Bemrich-Stolz C, Varni JW. 2014. PedsQL^{TM} Multidimensional Fatigue Scale in sickle cell disease: feasibility, reliability, and validity. Pediatr Blood Cancer 2014 61(1):171-7.Payne, K.A. et al., 2007. Clinical and economic burden of infused iron chelation therapy in the United States. *Transfusion*, 47(10):1820–9.

Porter, J., Bowden, D. & Economou, M., 2012. Health-related quality of life, treatment satisfaction, adherence and persistence in β-thalassemia and myelodysplastic syndrome patients with iron overload. Anemia 2012:297641-51.

Skevington SM, Lotfy M, O’Connell KA. 2004. The World Health Organization’s WHOQOL-BREF quality of life assessment: psychometric properties and results of the international field trial. A report from the WHOQOL group. Qual Life Res. 13(2):299-310.

Tomlinson D, Hinds PS, Ethier MC, Ness KK, Zupanec S, Sung L. 2013. Psychometric properties of instruments used to measure fatigue in children and adolescents with cancer: a systematic review. J Pain Symptom Manage. 45(1):83-91.
